# Machine Learning-Based Prognostic Prediction for Knee Osteoarthritis After High Tibial Osteotomy Using Wavelet-Derived Gait Features

**DOI:** 10.3390/jfmk11010094

**Published:** 2026-02-26

**Authors:** Koji Iwasaki, Kento Sabashi, Hidenori Koyano, Yuji Kodama, Shigeyuki Sakurai, Kengo Ukishiro, Ryusuke Ito, Hisashi Matsumoto, Yuichiro Abe, Noriaki Mori, Chiharu Inoue, Yasumitsu Ohkoshi, Tomohiro Onodera, Eiji Kondo, Norimasa Iwasaki

**Affiliations:** 1 Department of Functional Reconstruction for the Knee Joint, Faculty of Medicine, Hokkaido University, Sapporo 060-8638, Japan; 2 Department of Rehabilitation, Hokkaido University Hospital, Sapporo 060-8648, Japan; 3 Faculty of Medicine of Biomedical Science and Engineering, Hokkaido University, Sapporo 060-0815, Japan; 4 Department of Rehabilitation, Wajokai Eniwa Hospital, Eniwa 061-1449, Japan; 5 Department of Rehabilitation, Hakodate Orthopedics Clinic, Hakodate 041-0802, Japan; 6 Department of Rehabilitation, Hokushin Orthopaedic Hospital, Sapporo 060-0908, Japan; 7 Sapporo Medical Research LLC, Sapporo 065-0013, Japan; menchi@athena.ocn.ne.jp; 8 Department of Orthopedic Surgery, Wajokai Eniwa Hospital, Eniwa 061-1449, Japan; 9 Department of Orthopedic Surgery, Hokushin Orthopaedic Hospital, Sapporo 060-0908, Japan; 10 Department of Orthopedic Surgery, Hakodate Orthopedics Clinic, Hakodate 041-0802, Japan; 11 Department of Orthopaedic Surgery, Faculty of Medicine and Graduate School of Medicine, Hokkaido University, Sapporo 060-8638, Japanniwasaki@med.hokudai.ac.jp (N.I.); 12 Center for Sports Medicine, Hokkaido University, Sapporo 060-8648, Japan; eijik@med.hokudai.ac.jp

**Keywords:** high tibial osteotomy, knee osteoarthritis, gait analysis, inertial measurement unit, machine learning, wavelet analysis, clinical outcome prediction

## Abstract

**Background**: Osteotomy around the knee (OAK) is a joint-preserving surgery for knee osteoarthritis, yet some patients experience suboptimal outcomes. Preoperative identification of high-risk patients remains challenging. This study aimed to develop a machine learning model to predict clinical outcomes after OAK using preoperative gait acceleration data from inertial measurement units (IMUs). **Methods**: This multicenter prospective study enrolled patients undergoing OAK. Preoperative gait was recorded using synchronized IMUs placed on the lumbar spine and tibia. Lumbar and tibial signals were used for gait-cycle segmentation, while wavelet-based time–frequency features were extracted from tibial acceleration only. Outcomes were defined by achievement of the minimal clinically important difference in ≥3 KOOS subscales at 2-year follow-up (Good vs. Poor). Continuous wavelet transform features (5–20 Hz) were summarized as mean and standard deviation across six stance subphases. A Random Undersampling Boost classifier was trained and evaluated using nested leave-one-subject-out cross-validation. A sensitivity analysis using logistic regression confirmed that the IMU-based prediction score was independently associated with outcome after adjustment for baseline KOOS (*p* = 0.047). **Results**: Of 67 enrolled patients, 37 were classified as Good and 30 as Poor outcome. For machine learning analysis, 1173 tibial acceleration gait-cycle waveforms were usable. The model achieved an AUC of 0.744 (95% CI, 0.610–0.860) using a median of 15 features (range, 5–25) with sensitivity of 0.69 and specificity of 0.72. The most informative predictors were the mean magnitude in the 5–8 Hz band during loading response (0–17%) and variability in the 5–8 Hz band during late stance (67–83%). No significant differences in baseline demographics or radiographic parameters were found between outcome groups. **Conclusions**: Preoperative IMU-derived gait acceleration features showed moderate-to-good discrimination between outcome groups and may support preoperative risk stratification and individualized perioperative management.

## 1. Introduction

Osteotomy around the knee (OAK) is a well-established joint-preserving surgery for knee osteoarthritis (KOA) [1], with numerous studies reporting favorable clinical outcomes [2,3]. However, a subset of patients remains dissatisfied with the results, and some even require conversion to total knee arthroplasty (TKA) due to disease progression [4]. Preoperatively identifying patients at high risk for such poor outcomes could significantly enhance patient satisfaction by enabling more informed surgical decision-making and the optimization of perioperative rehabilitation protocols.

Previously reported risk factors for poor outcomes after OAK include obesity, severe preoperative OA grade, and residual varus malalignment or valgus overcorrection [5,6]. However, these are primarily static clinical or radiological indicators that serve as risk factors at the population level; their utility in predicting outcomes for individual patients remains limited. Since OAK aims to preserve the native joint and modify the mechanical loading environment rather than replace the articular surfaces, postoperative outcomes are likely to be more sensitive to patient-specific physical function and dynamic loading patterns [7]. Therefore, dynamic functional assessments performed preoperatively may offer superior predictive value for clinical outcomes.

In recent years, gait analysis using inertial measurement units (IMUs) has emerged as a versatile tool for obtaining objective biomechanical metrics, such as trunk movement asymmetry [8] and the external knee adduction moment (KAM) [9,10,11]. These metrics have been shown to correlate closely with KOA severity and clinical symptoms [8]. In the field of TKA, it has been reported that preoperative IMU-based gait parameters are associated with postoperative functional outcomes [12]. Furthermore, the integration of machine learning algorithms has enabled the development of highly accurate models for predicting postoperative prognosis in TKA patients [13]. However, to our knowledge, no studies have yet utilized preoperative IMU gait data to predict clinical outcomes specifically for OAK.

The purpose of this study was to develop a machine learning-based system to predict the success or failure of clinical outcomes after OAK using preoperative IMU gait acceleration data. We hypothesized that preoperative acceleration patterns—specifically features reflecting dynamic stability during the loading response and stance phases—can predict the postoperative clinical prognosis.

## 2. Materials and Methods

### 2.1. Study Design and Participants

This multicenter prospective study included patients who underwent proximal tibial osteotomy for varus knee at participating institutions between 2020 and 2024. The study protocol was approved by the institutional review boards of all participating centers, and written informed consent was obtained from all participants.

Patients were eligible for inclusion if they were diagnosed with medial compartment KOA and underwent HTO surgery. The exclusion criteria were as follows: (1) Patients who did not undergo gait analysis using an IMU before and two years after the surgery. (2) Patients for whom the KOOS (Knee injury and Osteoarthritis Outcome Score) evaluation results before and two years after the surgery were not available. (3) Patients whose IMU sensor waveforms were disrupted, preventing accurate identification of the gait cycle. (4) Patients who underwent double-level osteotomy (DLO). We excluded DLO cases to ensure biomechanical homogeneity in our analysis, as the combined femoral and tibial corrections in DLO introduce distinct gait alterations compared to isolated tibial osteotomies.

The primary outcome was postoperative clinical success, classified based on changes in KOOS subscale scores between preoperative and 2-year postoperative assessments. Patient satisfaction was not assessed as a separate endpoint; outcomes were evaluated using KOOS, a widely used PROM in OAK research. Postoperative rehabilitation was not standardized across participating institutions and followed routine, site-specific clinical practice. Patients were classified into the Good outcome group if they demonstrated improvement exceeding the Minimal Clinically Important Difference (MCID) [14] in three or more of the five KOOS subscales, which included Pain, Symptoms, Activities of Daily Living, Sport/Recreation, and Quality of Life. Patients with improvement in two or fewer subscales were classified into the Poor outcome group. The objective of this study was to develop a classifier capable of predicting outcome group membership using only preoperative gait acceleration data.

Sample size was determined by data availability during the study period. Post-hoc power analysis indicated that with 67 patients (37 Good, 30 Poor), the study achieved 85% power to detect an AUC of 0.74 versus the null (AUC = 0.5) at α = 0.05.

### 2.2. Gait Data Acquisition

Gait acceleration data were collected using a wearable IMU system (LEOMO Inc., Tokyo, Japan). Each sensor unit incorporated a 3-axis accelerometer, 3-axis gyroscope, and magnetometer, with data sampled at 100 Hz and exported in CSV format. A key advantage of this system was that multiple sensor units were automatically synchronized in real time, ensuring precise temporal alignment of data. The system has been validated against marker-based three-dimensional motion capture in cycling [15,16]; however, direct validation during walking has not been performed for this specific device. The sensor specifications (3-axis accelerometer and gyroscope, 100 Hz) are comparable to those of commercially available IMU systems validated for gait analysis [17]. IMU sensors were attached to the lumbar back and the tibial tuberosity of the target leg using an elastic belt. The sensors were fixed as firmly as possible to prevent gait-induced vibrations from affecting the waveforms.

Participants performed level walking at their self-selected comfortable speed along a 10-m walkway (mean, 1.1 m/s; 95% CI, 1.0–1.3). Multiple trials were conducted to ensure adequate data collection. The coordinate system was defined such that ACC_X represented mediolateral acceleration, ACC_Y represented vertical (superior–inferior) acceleration, and ACC_Z represented anteroposterior acceleration. Angular velocities about the respective axes were denoted as GYR_X, GYR_Y, and GYR_Z.

### 2.3. Signal Processing and Gait Cycle Segmentation

All signal processing was performed using MATLAB R2025a (MathWorks, Natick, MA, USA). Raw acceleration signals were filtered using a 4th-order Butterworth low-pass filter with a 20 Hz cutoff frequency, applied bidirectionally using the filtfilt function to ensure zero-phase distortion.

Gait events were identified using a validated algorithm combining lumbar and tibial sensor data [18,19]. Initial contact was detected from the anterior peak of lumbar anteroposterior acceleration [18], while toe-off was detected from the anterior pitch peak of tibial angular velocity [19]. These events defined the stance phase of each gait cycle. To eliminate acceleration and deceleration effects, the first two and last two gait cycles of each walking trial were excluded from analysis.

Stance phase waveforms were time-normalized to 0–100% of stance duration and resampled to 101 points using spline interpolation. This normalization allowed for comparison across subjects with different walking speeds. A total of 1173 gait cycles from 67 patients were extracted for subsequent analysis.

### 2.4. Feature Extraction Using Wavelet Analysis

The resultant acceleration magnitude was first computed as the vector sum of the three orthogonal tibial acceleration components using the formula: Magnitude = √(ACC_X^2^ + ACC_Y^2^ + ACC_Z^2^) [20]. This composite signal represents the overall dynamic loading and movement pattern during stance. To capture the time–frequency characteristics of gait dynamics, we employed a systematic feature engineering approach based on wavelet decomposition [21,22]. The normalized stance phase (0–100%) was divided into six equal time slices: 0–17%, 17–33%, 33–50%, 50–67%, 67–83%, and 83–100%. The tibial acceleration magnitude signal (vector sum of three axes) was transformed using the continuous wavelet transform (CWT; MATLAB cwt function, analytic Morlet wavelet) with frequency limits of 5–20 Hz. Wavelet coefficients were summarized within five frequency bands (5–8, 8–11, 11–14, 14–17, and 17–20 Hz). For each of the 30 time–frequency regions (6 time slices × 5 bands), two complementary summary features were computed from the absolute coefficients—the mean (Mean), reflecting average coefficient magnitude (overall intensity), and the standard deviation (SD), reflecting within-region variability—yielding 60 features per gait cycle [21,22].

### 2.5. Machine Learning Model Development

A Random Undersampling Boost (RUSBoost) classifier [23] was employed to predict postoperative outcome group membership. RUSBoost is an ensemble learning method specifically designed for imbalanced datasets, combining random undersampling with adaptive boosting to address class imbalance issues.

Model performance was evaluated using a nested leave-one-subject-out cross-validation (nested LOSO CV) framework [24] to ensure unbiased estimation of model generalizability. This approach consisted of two nested loops: an outer loop for performance evaluation and an inner loop for hyperparameter optimization. In the outer loop, each of the 67 patients was held out sequentially as an independent test subject, with the remaining 66 patients forming the training set. Critically, to prevent any information leakage, all preprocessing steps—including minimum Redundancy Maximum Relevance (mRMR) feature selection [25], z-score normalization, and ReliefF ranking [26]—were performed independently within each training fold using only the training subjects’ data. Although features were extracted at the gait-cycle level (i.e., one feature vector per gait cycle), cross-validation splitting was performed strictly at the subject level such that all gait cycles from the held-out subject were reserved exclusively for testing in each outer fold. The inner loop employed a second LOSO CV within the training set to determine the optimal number of features (K) that maximized the Area Under the Curve (AUC) for distinguishing outcome groups. For each candidate K value (tested at K = 7, 13, 19, and 25, followed by refinement around the best value), predictions were generated for each inner-held-out subject and pooled to calculate a single AUC metric. The K value yielding the highest inner-loop AUC was then used to train the final model on the complete outer training set, which was subsequently evaluated on the outer-held-out subject. This nested structure ensured that K selection was based solely on training data, preventing optimistic bias in performance estimates.

Feature selection was performed using a two-stage approach within each training fold of the LOSO CV procedure. First, the initial 60 features were reduced to 30 using the mRMR algorithm, which selects features that have high relevance to the outcome while minimizing redundancy among selected features. Second, the ReliefF algorithm was applied to the 30 mRMR-selected features to assign importance weights based on their ability to distinguish between classes while accounting for feature interdependencies. To determine the optimal model complexity, the number of selected features (K) was treated as a hyperparameter and systematically varied from 5 to 25. The optimal K value was identified as the one that maximized the Area Under the Receiver Operating Characteristic Curve (AUC) across all cross-validation folds.

For each trained model, predicted probabilities were obtained at the gait-cycle level. To produce a single subject-level prediction for each held-out subject, gait-cycle-level predicted probabilities were aggregated within that subject using the median probability across all available gait cycles (primary aggregation method). All primary performance metrics (AUC, sensitivity, specificity, and accuracy) were computed at the subject level using these aggregated subject-level predictions.

The overall predictive performance of the final model was quantified using the AUC, with sensitivity, specificity, and accuracy also reported. Feature importance was visualized using heatmaps to identify the specific time–frequency regions most discriminative of postoperative outcomes. Feature importance heatmaps were generated post hoc for interpretability using ReliefF weights; these visualizations were not used for model selection or performance evaluation.

Sensitivity analysis for baseline dependency. To assess potential baseline dependency and ceiling effects related to the MCID-based outcome definition, we performed a subject-level logistic regression sensitivity analysis. The dependent variable was outcome group (Good vs. Poor), and covariates included the aggregated subject-level prediction score (median predicted probability across gait cycles) and baseline KOOS total (defined as the mean of the five KOOS subscales). Inference was based on likelihood-ratio tests.

### 2.6. Statistical Analysis

Baseline demographic and clinical characteristics were compared between the Good and Poor outcome groups using independent t-tests for continuous variables and chi-square tests for categorical variables. Statistical significance was evaluated using likelihood-ratio tests. Statistical significance was set at *p* < 0.05. All statistical analyses were performed using MATLAB R2025a and JMP Pro 17 (SAS Institute Inc., Cary, NC, USA).

## 3. Results

### 3.1. Patient Demographics and Baseline Characteristics

**Figure 1:**
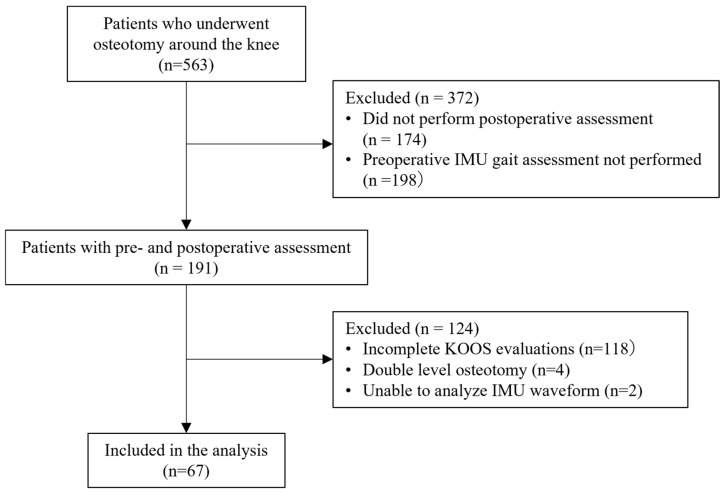
Flow chart.

A total of 563 patients underwent osteotomy around the knee during the study period. Following the application of exclusion criteria, 67 patients were included in the final analysis. Among the excluded patients, 372 were excluded due to missing IMU gait analysis data at either the preoperative or postoperative time point, 118 were excluded due to incomplete KOOS evaluations, 4 were excluded because they underwent DLO and 2 were excluded because gait cycles could not be accurately identified due to corrupted IMU sensor waveforms. Of the 67 included patients, 37 were classified into the Good outcome group and 30 into the Poor outcome group. A total of 1173 gait cycles were extracted across all subjects (Figure 1).

There were no significant differences between the Good and Poor outcome groups in terms of age, sex distribution, BMI, type of osteotomy, or radiographic leg alignment parameters including hip–knee–ankle angle (HKA), medial proximal tibial angle (MPTA), and mechanical lateral distal femoral angle (mLDFA) (all *p* > 0.05) (Table 1). Preoperative KOOS scores in the Good outcome group were significantly lower than those in the Poor outcome group across all five subscales (all *p* < 0.05). At the 2-year postoperative assessment, the Good outcome group demonstrated significantly higher scores than the Poor outcome group in four of the five KOOS subscales: Pain, Symptoms, Activities of Daily Living, and Sport/Recreation (all *p* < 0.05). The Quality of Life subscale showed no significant difference between groups at follow-up. Postoperative leg alignment parameters did not differ significantly between the two groups (all *p* > 0.05) (Table 2).

### 3.2. Predictive Performance of the Machine Learning Model

**Figure 2:**
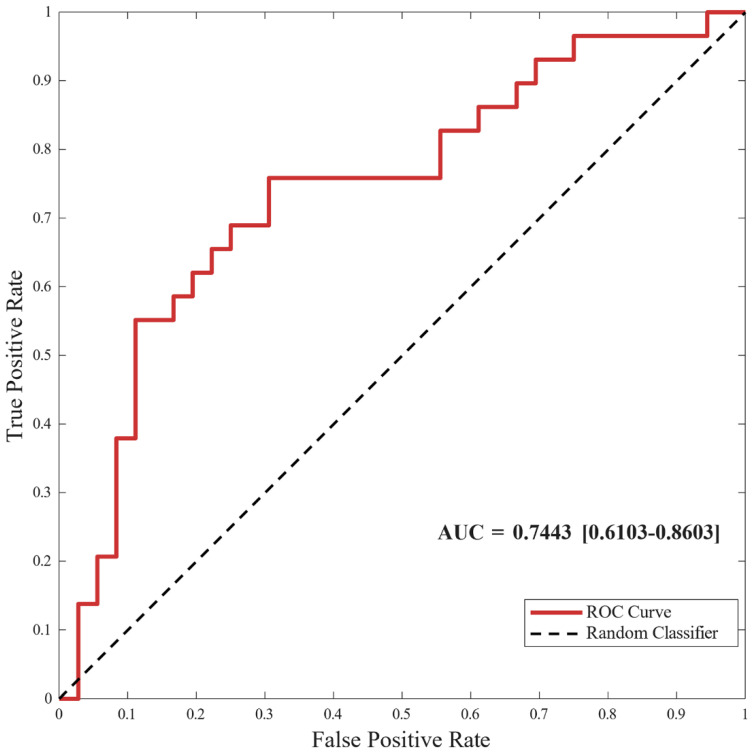
ROC curve for predicting clinical outcomes. The receiver operating characteristic (ROC) curve shows the subject-level predictive performance of the RUSBoost classifier evaluated using nested leave-one-subject-out cross-validation. The area under the curve (AUC) is 0.74 (95% CI: 0.61–0.86). The x-axis represents the false positive rate (1—specificity), and the y-axis represents the true positive rate (sensitivity). The diagonal dashed line indicates an AUC of 0.50.

The RUSBoost classifier’s predictive performance was evaluated using nested LOSO CV and achieved a subject-level AUC of 0.74 (95% CI: 0.61–0.86) (Figure 2). At the optimal threshold determined by Youden’s index, the model achieved a sensitivity of 0.69, specificity of 0.72, and overall accuracy of 0.71 (Table 3). The positive predictive value was 0.76 and the negative predictive value was 0.67.

The distribution of selected K values across the 67 outer folds showed a median of 15 features (mean: 16.8, range: 5–25). The most frequently selected values were K = 15 (22.1% of folds, n = 15) and K = 13 (20.9% of folds, n = 14). (Table 4).

### 3.3. Feature Importance Analysis

**Figure 3:**
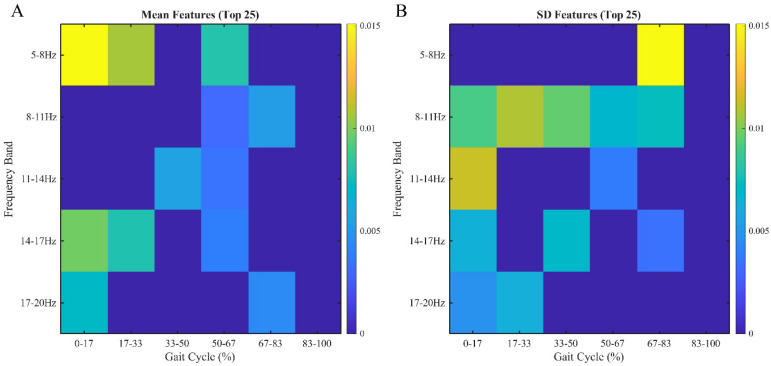
Heatmap of feature importance. The heatmaps display the relative importance of time–frequency features based on ReliefF weights for interpretability. Rows indicate frequency bands (5–20 Hz) and columns indicate stance-phase time windows (0–100%). (**A**) Mean features and (**B**) standard deviation (SD) features. Higher values indicate greater contribution. The most predictive regions were the Mean in the 5–8 Hz band during 0–17% of stance and the SD in the 5–8 Hz band during 67–83% of stance.

The contribution of each feature to the classification model was visualized using a heatmap based on ReliefF weights (Figure 3). The top 25 features identified by global ReliefF analysis were distributed across multiple frequency bands and time slices. The two features with the highest importance were the average magnitude (Mean) in the 5–8 Hz band during the 0–17% time window and the standard deviation (SD) in the 5–8 Hz band during the 67–83% time window.

For the Mean features, substantial contributions were observed in the 5–8 Hz band extending from the loading response through mid-stance, and in the 14–20 Hz band during the initial loading phase. For the SD features, the 8–11 Hz band showed contributions across a broad range, from initial contact through the late stance phase. Additionally, SD features in the higher frequency bands (8–20 Hz) were prominently distributed during the initial contact phase (0–17%).

Descriptive comparisons of the top-ranked wavelet-derived features showed generally higher variability (SD magnitude) in the Poor group during early stance (0–17%; 11–14 Hz and 14–17 Hz) and late stance (67–83%; 5–8 Hz), with a smaller difference in mean magnitude in the 5–8 Hz band during 0–17% (Supplementary Table S1).

### 3.4. Sensitivity Analysis

As a sensitivity analysis for potential baseline dependency of the MCID-based outcome definition, we fitted a subject-level logistic regression model including the aggregated subject-level prediction score and baseline KOOS total. The prediction score remained independently associated with outcome after baseline adjustment (OR = 0.13, 95% CI 0.01–0.98; likelihood-ratio test *p* = 0.047). Baseline KOOS total was also associated with outcome (OR = 0.91, 95% CI 0.86–0.95; *p* < 0.0001).

## 4. Discussions

This study developed a machine learning model to predict clinical outcomes after OAK using preoperative gait data, achieving an AUC of 0.74 (95% CI: 0.61–0.86). While previous studies have shown that sensor-based gait parameters can predict outcomes after total knee arthroplasty [12], few have examined joint-preserving surgeries like OAK using raw acceleration data. To our knowledge, this is the first study demonstrating that preoperative frequency-domain gait features can identify patients at risk of unsatisfactory results after OAK. The Good and Poor outcome groups showed no significant differences in baseline demographics, osteotomy type, or postoperative leg alignment, indicating that preoperative dynamic gait characteristics—rather than surgical technique or static correction—were decisive factors in clinical improvement.

The strongest predictor was the mean magnitude in the 5–8 Hz band during loading response (0–17%). Recent studies associate the 5–10 Hz band with impact loading during early stance [28,29]. Patients with abnormal acceleration patterns in this band during weight-bearing may have reduced capacity for impact absorption or knee stabilization. Since OAK relies on biological remodeling and cartilage unloading, preoperative deficits in shock absorption may limit functional recovery despite successful alignment correction.

Standard deviation in the 5–8 Hz band during late stance (67–83%) was also highly predictive. SD represents temporal variability and reflects gait variability, which is linked to impaired neuromuscular control in knee OA [30,31]. Late stance requires stable weight transfer; increased variability suggests compromised dynamic control or compensatory strategies. SD features in the 8–11 Hz band across 0–83% of stance further contributed to prediction, indicating that variability throughout stance may reflect overall gait instability. Notably, in our approach, predictions were generated at the gait-cycle level and then aggregated within each subject (median probability across gait cycles) to obtain subject-level predictions; thus, the reported performance reflects subject-level discrimination rather than within-subject cycle-to-cycle differences.

SD features in the 8–20 Hz band were prominent during initial contact (0–17%). Studies in running associate the 9–20 Hz range with impact-related accelerations [28,32]. Although these associations are established primarily in running, the interpretation of higher-frequency oscillations during loading in walking should be considered hypothesis-generating rather than definitive. Variability in this range may reflect inconsistent impact loading; however, the specific biomechanical meaning and clinical implications for OAK require validation using synchronized biomechanical measurements in walking. Descriptive comparisons showed that the Poor group tended to exhibit higher variability (SD) in higher-frequency bands during early stance (0–17%) and higher variability in the 5–8 Hz band during late stance, supporting the notion that phase-specific instability/impact-related signatures may be associated with poorer postoperative improvement. Future studies should validate the biomechanical meaning of these wavelet-derived time–frequency features in walking by synchronizing IMU signals with kinetics/kinematics (e.g., ground reaction forces and joint moments) and, ideally, confirming findings in external cohorts. These findings may suggest opportunities for targeted intervention. High-risk patients identified by the model could receive structured gait retraining, as biofeedback-based programs have reduced knee loading in OA populations [33,34]. Programs targeting excessive early-stance impact and core stabilization may help modify compensatory patterns. IMU-based gait assessment is increasingly used in OA management [17], supporting integration into routine preoperative screening.

Compared with prior IMU approaches that mainly estimate spatiotemporal gait parameters from wearable sensors, our approach leverages raw tibial acceleration to derive time–frequency features, enabling the capture of phase-specific and higher-frequency gait signatures that are not well represented by conventional summary metrics [35]. Tibial acceleration during walking has been associated with impact-related loading characteristics (e.g., vertical load rates) and knee pain in individuals with knee osteoarthritis, supporting the relevance of retaining higher-frequency information [36]. In addition, recent work has demonstrated that loading rate can be assessed in the frequency domain using accelerometry by separating impact-related and active-motion components across frequency bands, which conceptually aligns with our time–frequency feature engineering strategy [29].

This study has several limitations. First, external validation has not been performed. Although the present feature-engineering framework is compatible with routine clinical IMU recordings, generalisability across institutions and IMU systems will depend on harmonised acquisition protocols (e.g., sensor placement, sampling rate, and filtering) and may require calibration and/or domain-adaptation strategies. In addition, postoperative rehabilitation was not standardised across participating institutions, which may have influenced clinical outcomes and thus could affect the observed model performance and generalisability. Second, the sample size was modest (67 patients) with a relatively wide confidence interval (0.61–0.86), limiting generalizability. Third, we used only acceleration features without incorporating other variables (strength, radiographic morphology, psychosocial factors) that may improve prediction. Fourth, our MCID-based outcome definition may be influenced by baseline KOOS values and ceiling effects, as participants with higher baseline scores may have less room to improve. However, in a baseline-adjusted logistic regression sensitivity analysis, the aggregated subject-level prediction score remained independently associated with outcome after adjustment for baseline KOOS total; future studies should test alternative outcome definitions (e.g., absolute postoperative KOOS thresholds or residualized change scores) and perform external validation. Fifth, although the IMU system used in this study has been validated against optical motion capture in cycling [15,16], direct validation during walking has not been performed for this specific device; however, its sensor specifications are comparable to those of systems validated for gait analysis. Finally, frequency-domain interpretations were informed by running studies [28,32], which may not fully translate to walking. Despite these limitations, the nested cross-validation framework minimized bias. Future studies should expand the cohort, perform external validation, and confirm feature importance in independent datasets.

## 5. Conclusions

This study demonstrated that a machine learning model using preoperative IMU-based gait acceleration features predicted clinical outcomes after osteotomy around the knee with good discrimination (AUC 0.74; 95% CI: 0.61–0.86). The most informative predictors were the mean magnitude in the 5–8 Hz band during loading response (0–17%) and the variability (SD) in the 5–8 Hz band during late stance (67–83%), suggesting potential utility for preoperative risk stratification and targeted rehabilitation.

## Figures and Tables

**Table 1:** Baseline and postoperative characteristics.

	Baseline Characteristics			Postoperative Characteristics		
	Good (n = 37)	Poor (n = 30)	*p* Value	Good	Poor	*p* Value
Age (year)	61.8 (58.8–64.7)	60.3 (57.0–63.7)	0.51	N/A	N/A	N/A
Sex (Male:Female)	18:19	14:16	0.99	N/A	N/A	N/A
BMI (kg/m^2^)	27.2 (25.9–28.4)	27.1 (25.6–28.5)	0.92	27.3 (25.9–28.9)	27.6 (25.9–29.5)	0.89
OA grade (2:3:4)	10:22:5	9:15:6	0.70	9:23:5	8: 16: 6	0.68
HKA (degree)	−5.7 (−7–−4.2)	−6.2 (−7.8–−4.6)	0.34	2.8 (1.9–3.7)	2.5 (1.5–3.6)	0.69
MPTA (degree)	84.6 (83.6–85.6)	83.9 (82.8–85.1)	0.38	89.7 (88.6–90.8)	89.3 (88.0–90.7)	0.65
mLDFA (degree)	88.5 (87.7–89.4)	87.6 (86.7–88.6)	0.18	89.0 (87.3–90.1)	88.5 (87.1–89.5)	0.61
Type of osteotomy (OWHTO:OWDTO:CWHTO)	21:11:5	18:10:2	0.84	N/A	N/A	N/A
Walking speed (m/s)	1.1 (1.0–1.2)	1.2 (1.0–1.4)	0.43	N/A	N/A	N/A

BMI, Body mass index; OA grade, Kellgren Lawrence OA grade at medial compartment [27], HKA, Hip–knee–ankle angle; MPTA, medial proximal tibial angle; mLDFA, mechanical lateral distal femoral angle; OWHTO, open-wedge HTO; OWDTO, open-wedge distal tuberosity osteotomy; CWHTO, closed-wedge HTO; N/A, Not applicable.

**Table 2:** Baseline, postoperative and change in KOOS.

KOOS	Baseline Characteristics			Postoperative Characteristics			Changes Between Baseline and Postoperative Value		
	Good (n = 37)	Poor (n = 30)	*p* Value	Good	Poor	*p* Value	Good	Poor	*p* Value
Pain	47.9 (42.4–53.4)	62.8 (56.6–69.1)	<0.01	81.6 (76.5–86.7)	66.5 (60.7–72.3)	<0.01	34.1 (29.0–39.2)	2.8 (−3.1–8.6)	<0.01
Symptom	52.9 (47.2–58.4)	72.7 (66.3–79.0)	<0.01	80.8 (76.4–85.1)	70.5 (65.6–75.5)	<0.01	28.2 (23.4–33.0)	−2.9 (−8.5–2.6)	<0.01
ADL	40.3 (33.5–47.2)	65.1 (57.2–72.9)	<0.01	74.3 (67.6–81.0)	60.7 (53.0–68.3)	<0.01	34.2 (28.7–39.6)	−4.9 (−11.2–1.3)	<0.01
Sports/Rec	40.4 (32.3–48.5)	56.1 (46.8–65.3)	0.01	69.4 (61.3–77.5)	56.5 (47.2–65.7)	0.04	28.8 (23.4–34.2)	0.5 (−5.7–6.7)	<0.01
QOL	26.6 (21.1–32.1)	42.9 (36.7–49.1)	<0.01	61.1 (54.7–67.4)	51.7 (44.4–58.9)	0.06	34.5 (28.0–41.0)	8.6 (1.2–16.1)	<0.01

**Table 3:** Confusion Matrix and Classification Performance.

Confusion Matrix			
	Predicted Good	Predicted Poor	Total
Actual Good	26 (TP)	11 (FN)	37
Actual Poor	8 (FP)	22 (TN)	30
Total	34	33	67
**Classification Performance Metrics**			
	**Value**	**Calculation**	
Sensitivity (Recall)	0.70	26/37	
Specificity	0.73	22/30	
Positive Predictive Value (PPV)	0.76	26/34	
Negative Predictive Value (NPV)	0.67	22/33	
Accuracy	0.72	48/67	
F1-score	0.73	2 × (PPV × Recall)/(PPV + Recall)	

Classification threshold was determined by Youden’s index on the receiver operating characteristic (ROC) curve. TP, True Positive; TN, True Negative; FP, False Positive; FN, False Negative.

**Table 4:** Distribution of Optimal Feature Numbers (K) Selected Across 67 Nested Cross-Validation Folds.

K Value	Number of Folds	Percentage (%)
5	4	6.0
7	2	3.0
11	3	4.5
13	14	20.9
15	15	22.4
17	2	3.0
19	6	9.0
21	7	10.4
23	3	4.5
25	11	16.4

Note: K values were independently selected for each outer cross-validation fold through an inner leave-one-subject-out loop that maximized AUC.

## Data Availability

The original contributions presented in this study are included in the article/Supplementary Materials. Further inquiries can be directed to the corresponding author.

## Supplementary Materials

The following supporting information can be downloaded at: https://www.mdpi.com/article/10.3390/jfmk11010094/s1, Table S1: Descriptive comparisons of key wavelet-derived features between outcome groups.

## Abbreviations

**Abbreviation**	**Full Term**
ADL	Activities of Daily Living
AUC	Area Under the Curve
BMI	Body Mass Index
CI	Confidence Interval
CWHTO	Closed-Wedge High Tibial Osteotomy
CWT	Continuous Wavelet Transform
DLO	Double-Level Osteotomy
HKA	Hip–Knee–Ankle angle
HTO	High Tibial Osteotomy
IMU	Inertial Measurement Unit
KAM	Knee Adduction Moment
KOA	Knee Osteoarthritis
KOOS	Knee injury and Osteoarthritis Outcome Score
LOSO CV	Leave-One-Subject-Out Cross-Validation
MCID	Minimal Clinically Important Difference
mLDFA	Mechanical Lateral Distal Femoral Angle
MPTA	Medial Proximal Tibial Angle
mRMR	minimum Redundancy Maximum Relevance
OA	Osteoarthritis
OAK	Osteotomy Around the Knee
OWDTO	Open-Wedge Distal Tuberosity Osteotomy
OWHTO	Open-Wedge High Tibial Osteotomy
QOL	Quality of Life
ROC	Receiver Operating Characteristic
RUSBoost	Random Undersampling Boost
SD	Standard Deviation
TKA	Total Knee Arthroplasty
