# Supplementary material for: Machine Learning-Based Prognostic Prediction for Knee Osteoarthritis After High Tibial Osteotomy Using Wavelet-Derived Gait Features
Source: J Funct Morphol Kinesiol. 2026 Feb 26;11(1):94. doi: 10.3390/jfmk11010094 (PMC13027576; doi:10.3390/jfmk11010094)
Supplement: Supplementary file 1 [file jfmk-11-00094-s001.zip › jfmk-4102346-supplementary.pdf]

Supplementary Table S1. Descriptive comparisons of key wavelet-derived features between outcome groups.

Feature	Good (mean ± SD)	Poor (mean ± SD)	Effect size (Cohen's d, Poor–Good)
Mean magnitude (5–8 Hz), 0–17% stance phase	0.119 ± 0.053	0.144 ± 0.091	0.34
SD of magnitude (5–8 Hz), 67–83% stance phase	0.015 ± 0.005	0.019 ± 0.009	0.50
SD of magnitude (14–17 Hz), 0–17% stance phase	0.031 ± 0.016	0.041 ± 0.018	0.57
SD of magnitude (11–14 Hz), 0–17% stance phase	0.034 ± 0.014	0.050 ± 0.021	0.86

Values are subject-level summaries; for each subject, gait-cycle–level feature values were aggregated using the median across all available gait cycles. Cohen's d is reported as Poor–Good (positive values indicate higher values in the Poor group).
